# A hybrid CNN–SVM classifier for weed recognition in winter rape field

**DOI:** 10.1186/s13007-022-00869-z

**Published:** 2022-03-12

**Authors:** Tao Tao, Xinhua Wei

**Affiliations:** 1 Key Laboratory of Modern Agricultural Equipment and Technology, Ministry of Education/College of Agricultural Engineering, Jiangsu University, Zhenjiang, 212013 China; 2 Yangzhou Polytechnic Institute, Yangzhou, China

**Keywords:** Winter rape seeding, Weed recognition, Convolutional neural network, Support vector machine, Hybrid model

## Abstract

**Background:**

Weed recognition is key for automatic weeding, which is a challenging problem. Weed recognition is mainly based on different features of crop images. The extracted image features mainly include color, texture, shape, etc. The designed features depend on manual work, which is blind to some extent. Meanwhile these features have poor generalization performance on a sample set. The final discrimination results tend to have a greater difference. The current study proposed a deep convolutional neural network (CNN) with support vector machine (SVM) classifier which aims to improve the classification accuracy of winter rape seeding and weeds in fields.

**Results:**

The VGG network model was adopted, which received a true color image (224 × 224 pixels) of rape/weed as the input. The proposed VGG-SVM model was able to identify rape/weeds with average accuracies of 99.2% in the training procedures and 92.1% in the test procedures, respectively. A comparative experiment was conducted using the proposed VGG-SVM model and five other methods. The proposed VGG-SVM model obtained a higher classification accuracy, greater robustness and real time.

**Conclusions:**

The VGG-SVM weed classification model proposed in this study is effective. The model can be further applied to the recognition of multi-sample mixed crop images in fields.

## Introduction

In China, rape is an important source of edible oil. China is one of the world’s largest oilseed rape-producing countries. Winter rape is seriously affected by grass damage, and the key period of control weeds is the seedling stage. Weeding is a timely work for crops in the seedling stage, which is a necessary condition for high yield [1]. At present, chemical weeding methods are mainly used for large-scale spraying of herbicides [2], which has the advantage of low implementation costs and can be applied on all kinds of farmland topographies. The biggest drawback is the large consumption of herbicides, which inevitably affects a large number of non-target organisms [3] and destroys balance in the agricultural biological environment [4]. Therefore, how to improve the efficiency of spraying herbicide is a hot topic in the field of modern agriculture. The key problem to be solved is how to realize accurate and fast recognition of crops and weeds. Therefore, it is of great significance to study methods for the accurate and rapid classification of rape and weeds.

In the current investigation, the crop/weed discrimination was mainly based on machine vision. Scholars have mainly adopted image-processing methods, which involve distinguishing all kinds of crop targets based on different characteristics of crop images. A traditional weed classification technique follows five key steps: image acquisition, image pre-processing, feature extraction, applying machine learning classifier and evaluation of the performance [5–7]. The extracted image features mainly include: color [8–11], texture [9, 11, 13, 15], shape [11, 16–18], geometrical and spatial feature [19], SURF features [20], edge [21], SIFT features [21], statistical features [22], etc. This kind of method can realize the effective recognition of crops and weeds. Sabzi et al. [9] designed a new expert system based on color and texture features to identify weeds in potato crops for accurate spraying. Chen et al. [10] proposed a method combining multi feature fusion and SVM to identify and detect the position of corn seedlings and weeds. Yang et al. [8] developed a classification mode based on color indices with a support sector data description. And the accuracy of maize and weed detection reached 96.74%. Yang [14] proposed a novel shape description approach named multiscale triangle descriptor MTD for plant species recognition. Le et al. [15] evaluated a novel algorithm, filtered Local Binary Patterns with contour masks and coefficient k (k-FLBPCM), for discriminating between morphologically similar crops and weeds. Bakhshipour et al. [16] integrated several shape features to establish a pattern for each variety of the plants. Sabzi et al. [11] used a machine vision prototype based on video processing and meta-heuristic classifiers to identify and classify potatoes and five weed species. Le et al. [13] realized the distinction between corn and single species of weeds on the basis of Local Binary Pattern (LBP) texture features and SVM. Hamuda et al. [17] developed an image processing algorithm to recognize cauliflower from weeds in different growth stages. Lastly, Raja et al. [23] developed a real-time weeding system where a robotic machine detected the weeds and used a knife to remove them.

In the above solution, artificially designed features is the key issue. The designed features have certain blindness, which depend on manual work. Meanwhile, these features had poor generalization performance on a sample set. The final discrimination results tended to have a greater difference because of the difference of the sample set. This kind of method can realize the effective recognition of crops and weeds. However, there are more varieties of rape and weeds in the field. The image features are diverse, and the background is complex. The existing weed detection methods are mainly based on experience. They are subject to sample and artificial subjectivity. Factors such as angle of view, illumination, background and occlusion may make the pixel values of the same kind of image very different and the pixel values of different kinds of images very similar, which makes it impossible for us to recognize the object in the image by specifying certain features explicitly. Moreover, the image-processing method has poor robustness and low accuracy, which can't meet the needs of practical field applications.

Compared with traditional methods, the new method using convolutional neural network is directly driven by the data. It can also realize self-study of expression relations, which is excellent for data representation of images. The convolutional neural network has achieved good results in handwriting character recognition [24], face recognition [25], and behavior recognition [26]. Deep learning also has some applications in agriculture [27], such as flower discrimination [28], fruit recognition [29], leaf counting [30], plant recognition [31], weed detection [32]. Convolutional neural networks can independently learn and extract each local feature of data through multi-layer convolution and pooling operations, and obtain more effective abstract feature mapping than explicit feature extraction methods. Additionally, deep learning can automatically learn the hierarchical feature representation of images [30]. It utilizes the deep structure to obtain the global features of the sample image and context information of sample images, which greatly reduces the error rate of image recognition. Gao et al. [33] developed a deep convolutional neural network based on the tiny YOLOv3 architecture for C. sepium and sugar beet detection. Zou et al. [34] developed the semantic segmentation algorithm with a simplified U-net to segment weeds from the soil and crops in images. Yu et al. [35] reported several deep convolutional neural network models that are exceptionally accurate at detecting weeds in bermudagrass. Olsen et al. [36] contributed image dataset of weed species from the Australian rangelands and demonstrated real time performance of the ResNet-50 architecture. Trong et al. [37] developed a novel classification approach via a voting method by using the late fusion of multimodal deep neural networks on the Plant Seedlings dataset.

This research aims to solve the problems of blindness and experientialism of manually designed features such as color, shape, and texture. To this end, we took the rape seedling and its associated weeds as the research object. The main contribution of this work is a new approach to rape/weed classification using a convolutional neural network method based on collected sample pictures of seedlings and weeds. Firstly, a feature extraction network of rape seeding and four weeds based on VGG Net was designed. Then, training and testing experiments were carried out on the image dataset of rape seeding and weeds. This paper also studied the influence of network parameters such as learning rate, batch size, and classifiers on feature extraction and classification performance. Ultimately, compared with artificial neural network (ANN) and SVM methods, our experimental results were analyzed and discussed. It can be seen that the proposed vision-based classification system by applying convolutional neural networks was effective and feasible. And the recognition accuracy is improved. An additional motivation for this study was to optimize the selection and dosage of herbicides automatically, which has practical and economic significance for herbicide spraying.

## Experiments

### Configuration introduction

TensorFlow is an excellent deep learning framework [43, 44], and we employed it in the present study. The running environment was configured, and the parameters of the detection model were set according to the capability of existing hardware devices, including image batch size, dimensions of input images and depth of the basic network. The test environment and the hardware and software were as follows: Windows 10 OS, Nvidia GTX 1080 GPU, Core i7-7700 CPU, processing frequency of 3.6 GHz, parallel computing framework of CUDA9.0 with a deep neural network acceleration library of cuDNN7.0, Python version 3.6.5, and 1 TB memory.

### Data collection

**Fig. 1:**
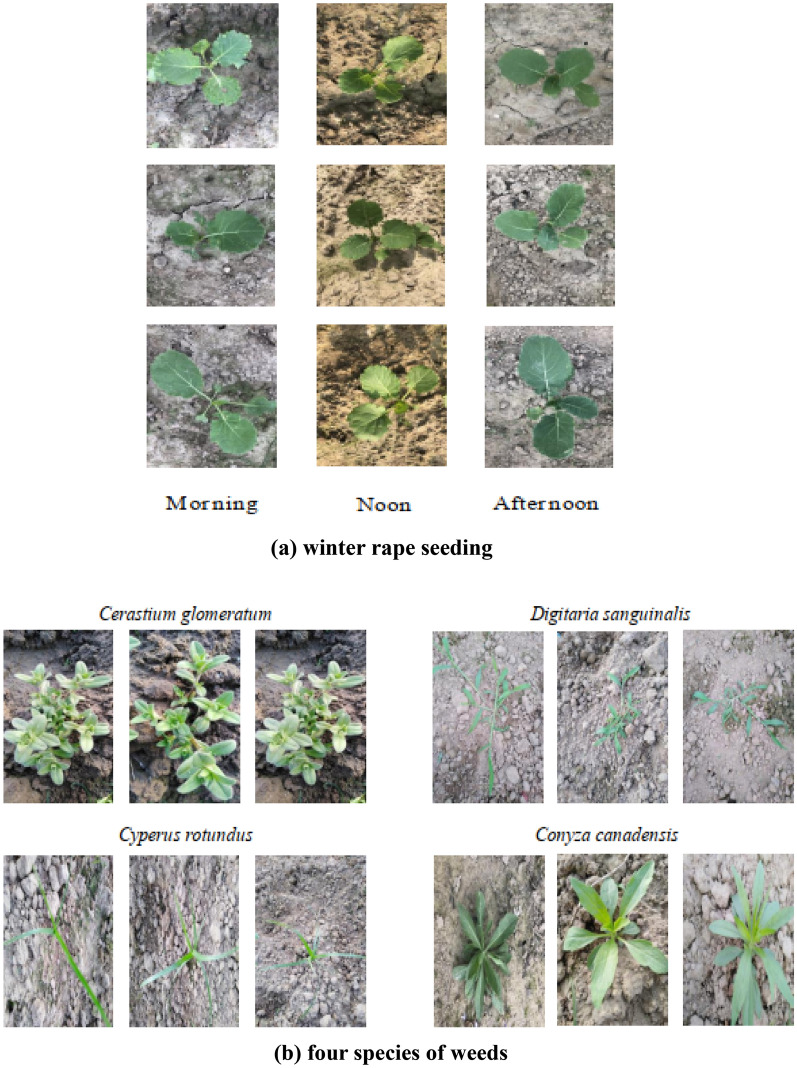
Examples of images of winter rape seeding and weeds. **a** winter rape seeding. **b** four species of weeds

In this study, winter rape seedlings and their associated weeds were used as research objects for classification experiments. The image acquisition work was completed in the experimental field of Jiangsu Agricultural Equipment Engineering College in January 2021. The acquisition equipment was the MV-VDM120SC industrial digital camera produced by Microvision Company. The main performance parameters were as follows: the sensor type was a CCD sensor of high-resolution. Images were 1280 × 960 pixels, the frame rate was 30 fps, and output was via a USB3.0 interface. The camera was placed 60 cm from the ground, and images were taken vertically. During the seedling growth of winter rape, four image acquisitions were carried out. The sample images were winter rape seeding (*Brassica campestris L.*) in the seedling stage and associated weeds: *Conyza canadensis* (L.) *Cronq*., *Digitaria sanguinalis* (L.) Scop., *Cerastium glomeratum* Thuill. and *Cyperus rotundus* L. Taking into account the possible environmental conditions during weeding, each image acquisition is performed under three different light conditions. In order to speed up the modeling process and improve recognition efficiency, the image size was compressed to 224 × 224 pixels. These images were used to build an image dataset, which ultimately contained 1500 images (700 rape seeding samples and 800 weeds samples). Images of winter rape at different time period and four species of weeds were used for model training and testing, as shown in Fig. 1.

In order to build the training set and test set of the model, the target categories of each sample image were marked, and the labels of each sample image were obtained. We randomly distributed the samples of rape seeding and weed with 80% for training and 20% for test. The performance of the recognition model was evaluated by fivefold cross-validation. All the image samples were divided into 5 sub-sample sets. Each sub-sample set included 300 sample images, including 140 rape seeding sample images and 160 weed sample images, which were random and didn’t duplicate the images in the dataset. Each sub-sample set (300 images) was used as the test set once, and the remaining 4 sub-sample sets were combined together (1200 images in total) to form the training set (80% for training, 20% for validation). In the experiment, the image of the training set was flipped horizontally, and the sample size of the training set was expanded to double the original. More samples participating in the training can reduce overfitting and strengthen the stability of the model [45].

### Minibatch

In general, in order to accurately calculate the gradient of the loss function versus a parameter, it is necessary to calculate each sample on the entire dataset. For a deep network, the calculation is very large. Therefore, in practice it is preferred to randomly sample a small number of samples from the dataset in batches, and then calculate the average over these batch samples. This sampling strategy is called minibatching, and the gradient descent method based on the minibatch is called random gradient descent. The algorithm [46] for stochastic gradient descent update parameters is as follows:

Choose an initial vector of parameters $w$ and learning rate $\upeta$

While failure to meet stop criteria do.

Random sampling samples $\left\{{x}^{\left(1\right)},{x}^{\left(2\right)},\dots ,{x}^{\left(m\right)}\right\}$ in the training set, target is ${y}^{\left(i\right)}$

Compute gradient estimation:$\mathrm{g}\leftarrow \frac{1}{m}{\nabla }_{\mathrm{w}}{\sum }_{i}L\left(f\left({x}^{\left(i\right)},w\right),{y}^{\left(i\right)}\right)$

Update:$$w\leftarrow w-\mathrm{\eta g}$$

end while.

Increasing the minibatch size within a reasonable range can effectively improve the efficiency of matrix parallelization and speed up data processing. Our experiment tested the accuracy of the model on the test set when the minibatch size was 32, 64, 128 and 256. The performance was measured using mean average precision (mAP) [6]. The results are shown in Table 1.

**Table 1:** Mean average precision (mAP) scores of classification model

Minibatch size	32	64	128	256
mAP	72.26	76.37	80.21	83.35

Table 1 shows the relationship between minibatch size and mAP. It can be seen that the larger the minibatch, the higher the mAP. However, the increment in mAP was not linear. For example, the increase in minibatch size from 64 to 128 and from 128 to 256 was doubled, but the corresponding mAP increased by about three percentage points. Since the training time increased obviously with the increase in minibatch size, the learning of parameters became slower, and training a small minibatch needed a smaller learning rate to maintain stability. When the minibatch size increased to a certain extent, the descending direction of the loss function tended to be stable. Based on the above, the candidate minibatch size was selected as 128.

### Learning rate and momentum

The choice of learning rate and momentum has a direct impact on the training speed and results of the detection network. If the learning rate is too high, the parameter update misses the optimal value, and it is easy to fall into the local extreme value, which will lead to the failure of training. If the setting is too small, it will lead to excessive training time. In this paper, some commonly used learning rates and momentum were selected as candidates to determine the appropriate learning rates and momentum through experiments. We selected 0.1, 0.0005, and 0.00001 as candidate values for the initial learning rate, and selected 0.5 and 0.9 as candidate values for momentum coefficient. We also selected 128 as candidate values for batch. Based on these candidate values, a series of comparative experiments were designed. The learning rate was determined, then the batch size was determined, and finally the momentum was determined. The combination of parameters and the test results are shown in Table 2.

**Table 2:** Accuracy of different parameter combinations

No	$\upeta$	α	mAP(%)
1	0.05	–	–
2	0.0005	–	82.31
3	0.00001	–	85.16
4	0.00005	–	89.62
5	0.00005	0.5	90.53
6	0.00005	0.9	92.38

**Fig. 2:**
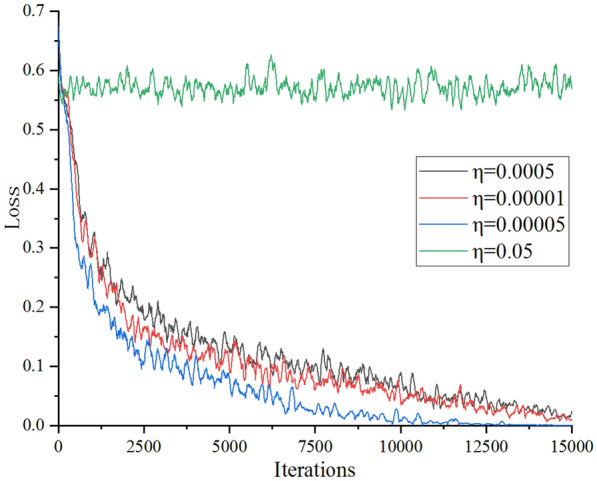
Loss curves under different learning rates

Without momentum, the influence of different learning rates on detection accuracy is tested on the basis of setting the batch number to 128. As shown in Fig. 2, when the learning rate was 0.05, the training diverged. The possible reason for this is that 0.05 as the initial learning rate is too large. When the learning rate was 0.0005, the mAP was slightly less than the corresponding value of 0.00001, which may have been due to the slow iteration of the loss function value of 0.0005 to the neighborhood of the minimum value relative to 0.00001 (Fig. 2). Accordingly, in the fourth group of experiments, 0.00005 was determined as the initial learning rate. Finally, the mAP of the model with momentum coefficients of 0.5 and 0.9 was tested by adding momentum alongside the fifth set of parameters. As can be seen from Table 2, the mAP of the model with momentum coefficients of 0.9 was the highest. Therefore, in order to train the VGG model, the initial learning rate was 0.00005, batch size was 128, and momentum coefficient was 0.9.

### SVM classifier

For the SVM classifier of the VGG-SVM model, we used the radial basis kernel function. We adopted the grid search to find out the optimal penalty parameter *C* and kernel parameter γ by applying the fivefold cross validation method. Their search ranges were *C* = [2^–8^, 2^–7^,…, 2^8^] and γ = [2^–10^, 2^–9^,…, 2^6^] with default values for other parameters. The final parameters were *C* = 2^4^ and γ = 2^–9^ then used to train the SVM classifier.

## Results and analysis

### VGG model test

**Fig. 3:**
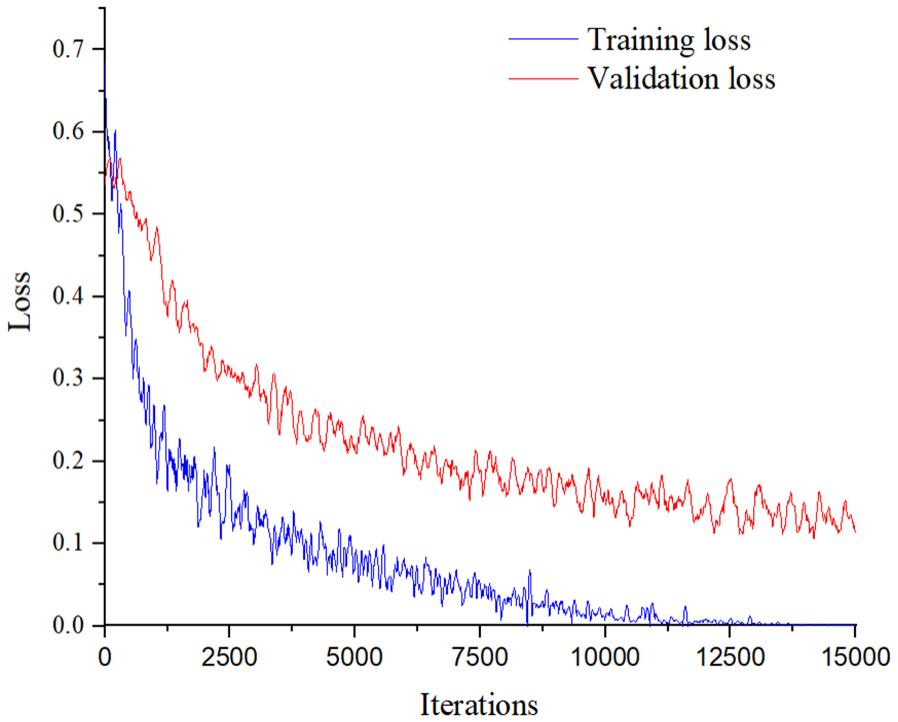
Loss curves

**Fig. 4:**
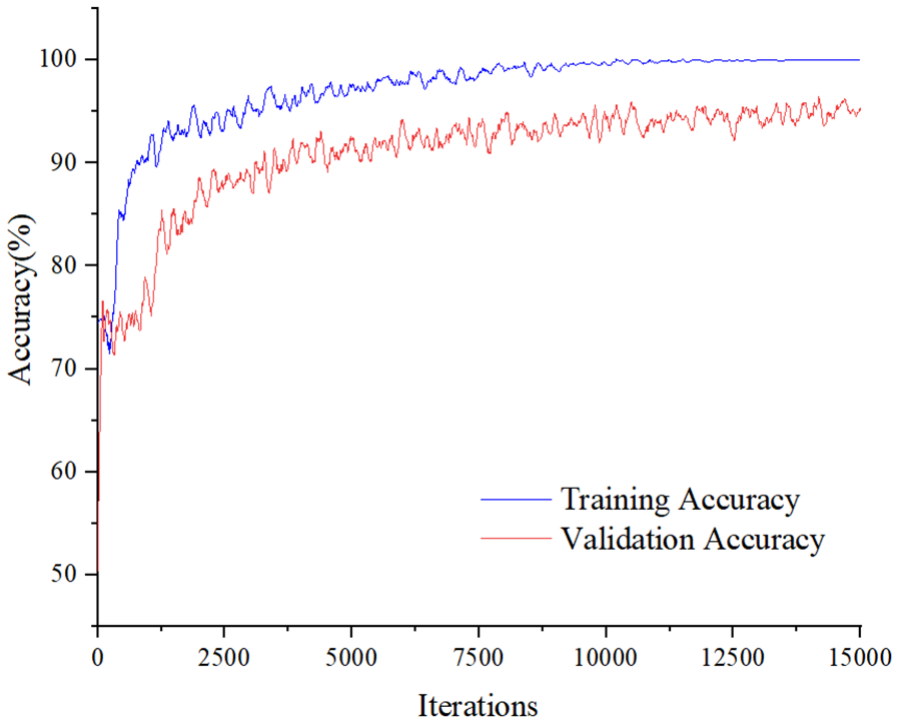
Accuracy curves

In this experiment, the minibatch size was 128, the learning rate was 0.00005, and the momentum coefficient was 0.9 (as determined in “Learning rate and momentum” section). The experimental results are shown in Figs. 3 and 4. Figure 3 shows the curve of training error and validation error varying with the number of iterations. It can be seen that with the increase in iterations, training error decreased, which indicated that the training state was good and no over-fitting state occurred. After 13,000 iterations, the training error tended to zero, which indicates that the gap between the output value and the real value of the network became smaller and smaller with the increase of iterations. Meanwhile, after 14,000 iterations, the validation error showed a stable fluctuation state, indicating that the gap between the output value and the real value of the network was stable during the validation process. Figure 4 also shows the change curve of the accuracy with the number of iterations. It can be seen that the accuracy of the training set was 0.72 at the first iteration, and was 100 after 11,000 iterations. In this process, the accuracy of the validation set was 0.51 at the first iteration and it declined slightly after 5000 iterations. The curve also shows slight fluctuations, and the accuracy finally reached 0.92. The final training recognition rate was 100%, and the average validation recognition rate was 91.2%.

### Evaluation of VGG-SVM model

The results of weed classification for test dataset were arranged in confusion matrices, including true positive (TP), true negative (TN), false positive (FP), and false negative (FN). In this experiment, TP represents the weeds that are correctly classified weeds; TN represents the rapes that are correctly identified as rapes; FP represents the rapes that are incorrectly classified as weeds; and FN represents weeds are incorrectly identified as rapes.

In order to evaluate performance, four common measures were calculated: accuracy, precision, recall, specificity. In this context, accuracy is the ratio of number of correct predictions (weeds) to the total number of test dataset samples; precision shows the ability of the model to accurately classify weeds; recall reflects the ability of the model to detect weeds. specificity measures the proportion of actual negatives that are correctly identified. All the above four measures are ranged from 0 to 1, high value means the good classification ability of the model, their definitions are as follows:

1 $$\mathrm{Accuracy}=\frac{\mathrm{TP}+\mathrm{TN}}{\mathrm{TP}+\mathrm{FP}+\mathrm{TN}+\mathrm{FN}}$$

2 $$\mathrm{Precision}=\frac{\mathrm{TP}}{\mathrm{TP}+\mathrm{FP}}$$

3 $$\mathrm{Recall}=\frac{\mathrm{TP}}{\mathrm{TP}+\mathrm{FN}}=\mathrm{TPR}$$

4 $$\mathrm{Specificity}=\frac{\mathrm{TN}}{\mathrm{TN}+\mathrm{FP}}=1-\mathrm{FPR}$$

**Fig. 5:**
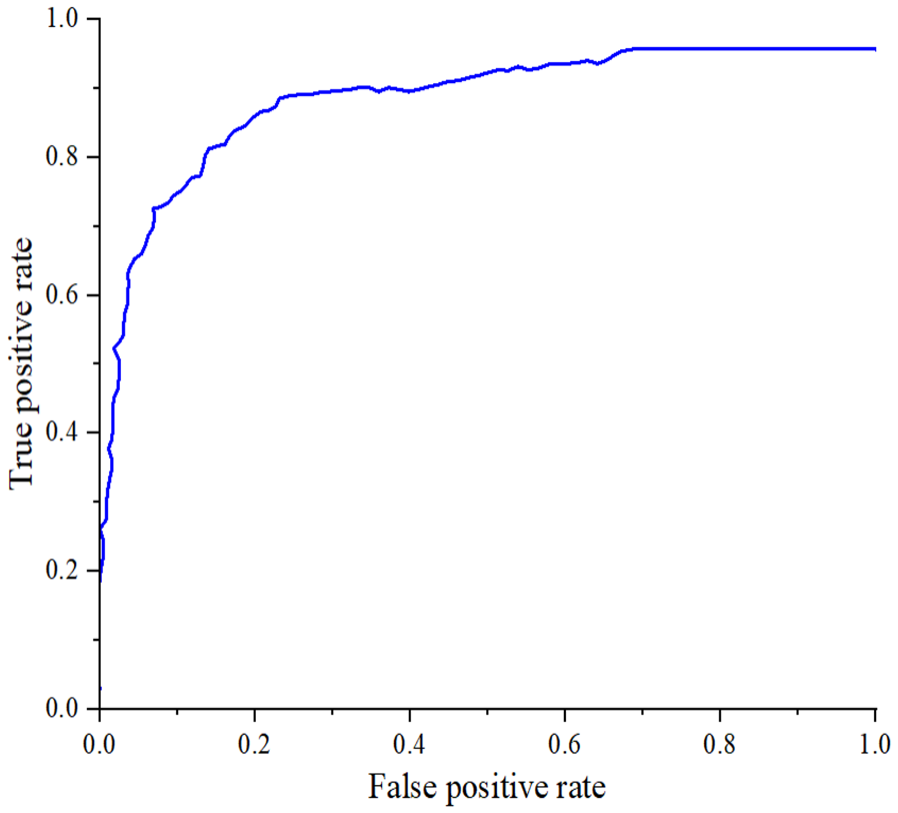
ROC curve

The receiver operating characteristic curve (ROC) reflects the relationship between sensitivity and specificity. The X-axis is 1-specificity, also known as false positive rate (FPR). The closer the FPR is to 0, the higher the accuracy. The Y-axis represents sensitivity, also known as true positive rate (TPR). The closer the TPR is to 1, the better the accuracy. In Fig. 5, the best boundary value of the classifier occurs when the FPR is 0.257 and the TPR is 0.881. That is, at this point, it is closest to the upper left corner, and the classification effect is optimal. According to the position of the curve, the whole graph (Fig. 5) is divided into two parts. The area under the curve is called AUC, which is used to indicate the prediction accuracy. The value of AUC is between 0 and 1. The higher the AUC value, that is, the larger the area under the curve, the higher the prediction accuracy. In Fig. 5, the value of AUC is 0.923. It shows that the VGG-SVM model has a good classification effect.

**Fig. 6:**
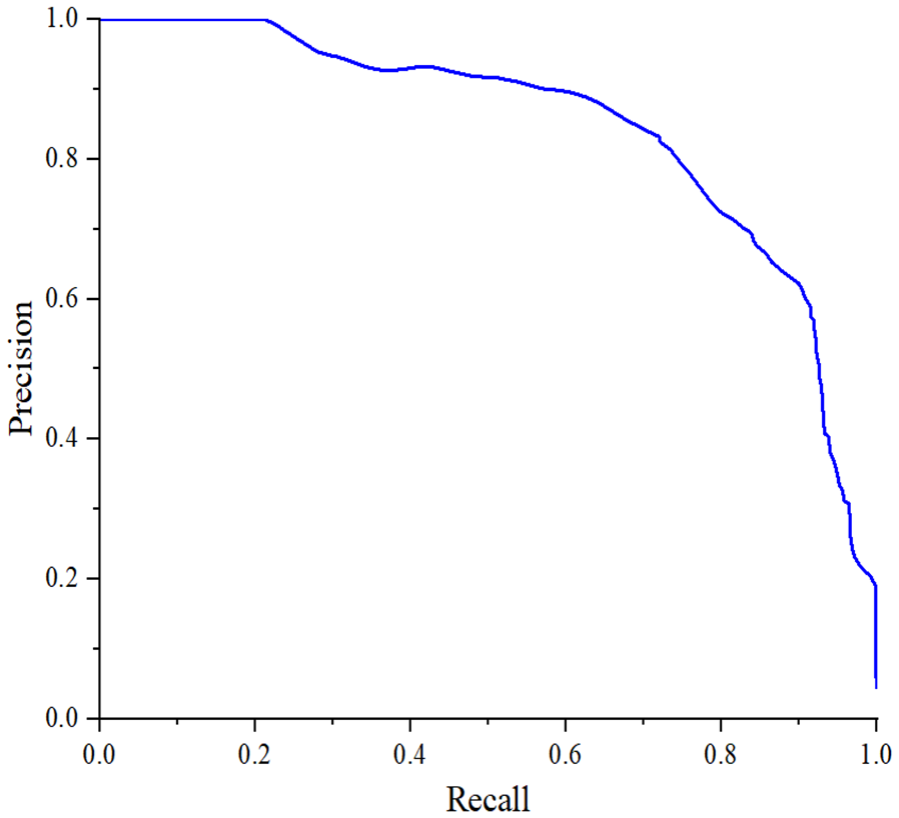
PR curves

The PR curve visually shows the recall and precision of the classifier in the test dataset sample. The X-axis is recall and the Y-axis represents precision. We can observe the change of value of precision and recall when the threshold changes. In Fig. 6, while the value of recall increases, the value of precision remains high. It indicates that the VGG-SVM classifier has a good performance.

### Comparing the performance of methodologies

In this section, we compare some state-of-the-art models in the current field. Recently, deep learning based methods has been widely applied in weed recognition. Yu et al. [35] adopted VGGNet as backbone for feature learning which has achieved competitive performance at detecting weeds in bermudagrass. Instead of using conventional features extracted from deep CNN, Bakhshipour et al. [16] employed ANN to optimize shape features and moment invariant features, which paied more attention on features with strong influence and lowering the threshold for model training. Besides, Olsen et. al [36] proposed a dataset consist of weed species from the Australian rangelands, and validated real time performance based on the ResNet-50 architecture. However, Trong et al. [37] argued that multi-modal features may be more conducive to weed recognition and developed a novel classification approach via a voting method by using the late fusion of multimodal deep neural networks, including NASNet, VGG, Resnet, Mobilenet. Moreover, Chen et al. [10] used the SVM classifier based multi feature fusion, including the histogram of oriented gradient feature, rotation invariant local binary pattern (LBP) feature, Hu invariant moment feature, Gabor feature, gray level co-occurrence matrix, and gray level-gradient co-occurrence matrix. We compared the above methods, and all the results are shown in Table 3.

**Table 3:** Accuracy performance analysis of methodologies

Method	VGG-SVM	VGG [35]	ANN [16]	SVM [10]	ResNet [36]	Trong et al. [37]
Test times	Accuracy (%)	Accuracy (%)	Accuracy (%)	Accuracy (%)	Accuracy (%)	Accuracy (%)
1	92.3	90.3	89	87.7	89.7	91.3
2	92.3	89.7	88	85.7	90.3	91
3	91.3	91.3	88.3	88	90	90.3
4	92	90.7	88.3	86.7	88.7	90
5	92.7	90.7	86	87.7	89.3	91.3
Mean	92.1	90.5	87.9	87.2	89.6	90.8
Std	0.52	0.59	1.13	0.87	0.62	0.60

Table 3 shows the proposed VGG-SVM has outperformed other models by a large margin. The average classification accuracy can achieve 92.7%. The VGG model and the ResNet model were similar in classification performance. Limited to the weak generalization ability of manually designed features, the performance of ANN and SVM are inferior to other deep learning based models. Meanwhile, the maximum classification accuracy of the VGG-SVM model was 92.7% and its minimum accuracy was 91.3%. The standard deviation of accuracy was 0.52%, which illustrated the classifier performance was stable. However, the stability of the ANN model and the SVM model was relatively poorer. The three kinds of deep neural networks had similar performance on stability. According to the data in Table 3, it can illustrate that the classification accuracy data of the VGG-SVM had a smaller spatial dispersion and less variation. Therefore, it had a stronger classification stability and more prominent generalization performance in practice.

Compared with the VGG model, the classification accuracy of the VGG-SVM was improved by 1.8%. The difference is mainly due to different optimization criteria. The softmax learning algorithm uses empirical risk minimization criterion (ERM) to minimize the prediction error of the training set. Minimizing empirical risk makes it easy to overfit the training set. Conversely, the SVM adopts structural risk minimization. The SVM aims to minimize the generalization error by using structural risk minimization principles for the test set. The structural risk minimization criterion is used to integrate the model complexity into the optimization objective, which solves the over-fitting problem and results in better generalization performance. As a result of a maximized margin, the generalization ability of the VGG-SVM model is greater than that of the VGG model.

### Comparing the performance under different light conditions

In order to verify the effectiveness and robustness of the proposed framework, we validated the performance of pre-trained model under three realistic lighting conditions. From the perspective of practical application, it was more reasonable to follow the laws of nature and choose the corresponding images according to the time period. In order to simulate reality, we randomly selected images taken in the morning, at noon, and in the afternoon separately as the test set to validate the existing model separately. Every test set consisted of 300 images taken from sources other than the original data set. For each test set, 5 experiments were conducted, and 200 pictures were randomly selected for each test (see Table 4).

**Table 4:** Accuracy performance under different light conditions

Period of time	Morning	Noon	Afternoon
Test times	Accuracy (%)	Accuracy (%)	Accuracy (%)
1	93	93	92
2	92	91.5	91.5
3	93	91	92
4	92.5	92	92.5
5	93	91.5	91.5
Mean	92.7	91.8	91.9
Std	0.45	0.76	0.42

The average performance under three light conditions was almost the same as the results of the VGG-SVM in Table 3, which demonstrate the proposed framework was not affected by the light conditions. The proposed model had great robustness for rape/weeds classification in field.

## Discussion

In this paper, a tailored framework for rape/weed classifier has been proposed, which is a vital step to support rape protection and prevent weed dispersal. The proposed model, which is consist of a VGG network for fine-grained feature learning and a SVM classifier for prediction, performed well in for identifying weeds. To validate the performance, we first created a novel dataset, which is specifically designed for rape/weed recognition, and then we conducted comprehensive experiments based on it. And the results of experimental show that the proposed model is capable of achieving desirable performance in identifying rape seeding and weeds. Besides, in order to simulate reality, we randomly select images taken in the morning, at noon, and in the afternoon separately to validate our model. However, the illumination condition is not efficiently labeled in the dataset. In future work, we will overcome the above shortcomings and employ variant sequence of images to better simulate the realistic field conditions. Moreover, our model will be transferred to other plant recognition and detection tasks for a more robust exploration.

## Conclusion

In this paper, the winter rape seedlings and four weeds were taken as research objects. To solve the problems of inaccurate recognition results based on artificially designed features and weak generalization ability of feature extraction, a convolution neural network weed recognition model was established. The proposed model achieved 99% training accuracy and 92% testing accuracy in identifying winter rape seedlings and weeds. The experimental results showed that the average classification accuracy of this method was 92.1% and the standard deviation was 0.52%. This method had excellent generalization performance and achieved stable and high classification accuracy. Compared with the other models, the classification accuracy of our method was improved by 1.4% at least. Moreover, the standard deviation was reduced, and the stability was improved. Furthermore, compared with the VGG model, the proposed model had higher classification accuracy. The VGG-SVM weed classification model proposed in this study was effective, which could obtain high recognition accuracy, stability, and real-time processing ability, which provides a useful reference for intelligent mechanical weeding. This paper only realizes the classification of single winter rape seedling/weed samples, and target recognition in multi-sample mixed images remains a problem to be solved in automatic weeding. Therefore, next, we will consider the classification and recognition of multi-sample mixed images.

## Proposed methodology

**Fig. 7:**
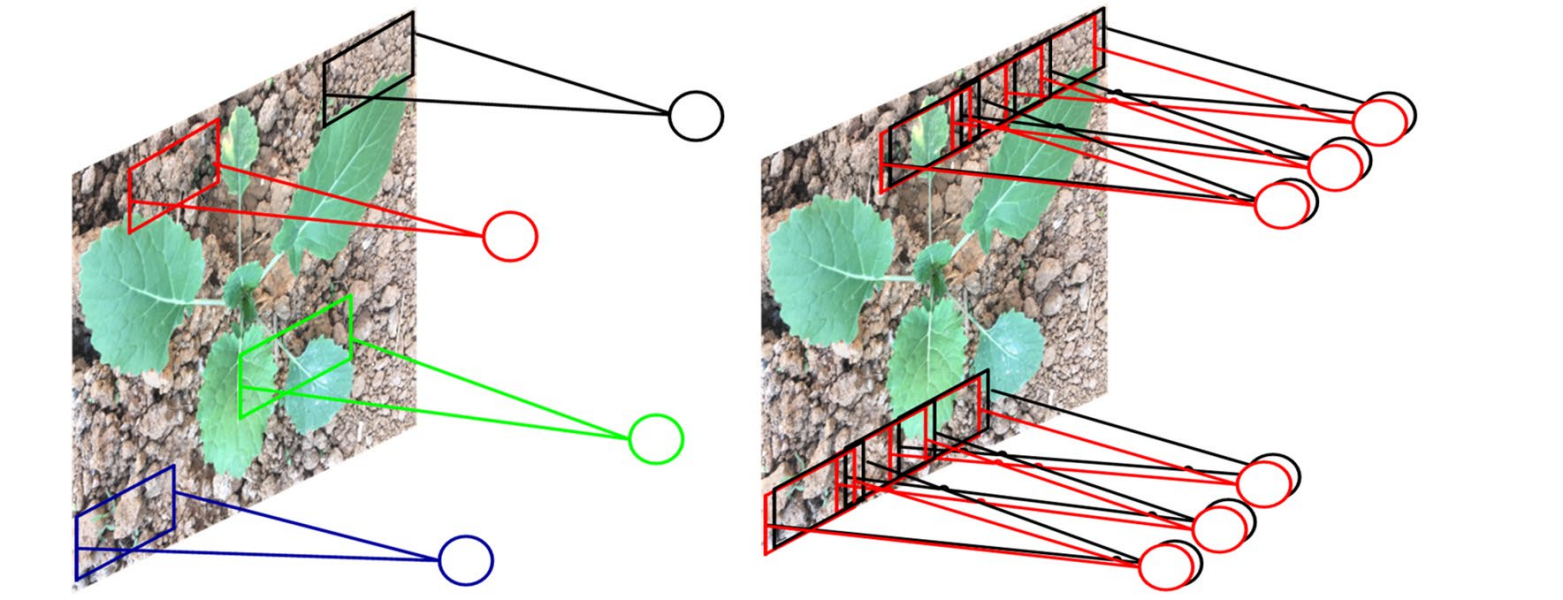
The neural net and the convolutional net

The CNN has achieved good classification results in the field of computer pattern recognition. The basic structure of the CNN is composed of an input layer, convolution layer, pooling layer, full connection layer, and output layer [38]. Several convolution and pooling layers are alternately set. The convolution layer is locally connected to the neuron. By choosing the weights, new parameters are calculated as the input of the next neuron. Such a convolution process can extract different features of different inputs. The difference between neural network and convolutional network is shown in Fig. 7.

The ability to artificially distinguish weeds and rape is limited, and the comparison of pixels in a local area can better distinguish their categories. In CNN network, each hidden layer node only needs to connect to a small local pixel in the image to obtain the local features of images; then, it can abstract the local features to get the global features at a higher level. Network depth can promote the reuse of each image feature, greatly reduce the number of weight parameters to be trained, and select more abstract features in high-level expression. It is difficult to distinguish weeds from rape because they are different not only in shape and texture, but also in color. Therefore, it is particularly important to increase the depth of the network and improve the performance of the network.

### VGG net

**Fig. 8:**
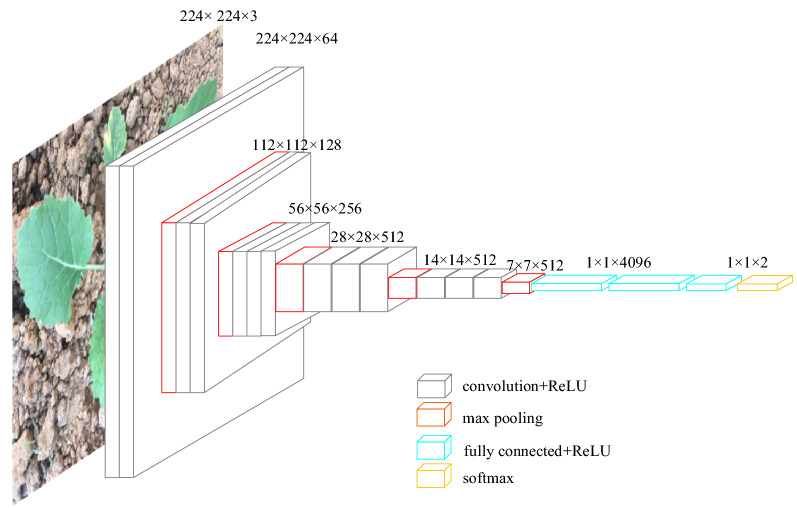
Structure of the VGG Net

On the basis of a single-layer convolution neural network, we increased the depth of the network. The VGG network model was adopted [39]. The network had 19 hidden layers, including 16 convolution layers and 3 full connection layers. The network also has two image feature layers and one classification feature layer. In order to reduce the number of network training parameters, a 3 × 3 size convolution was adopted in the whole convolution network. Three non-linear activation functions were used to increase the ability of non-linear expression. This made the segmentation plane more separable and reduced the number of parameters. Figure 8 shows the structure of the VGG network. The convolutional layer is designed to learn characteristics of the previous layer. Softmax provides a classification probability of the input to belong to any of the trained classes. Then, the category with the highest probability is considered the predicted classification label.

### Spatial pyramid pooling

**Fig. 9:**
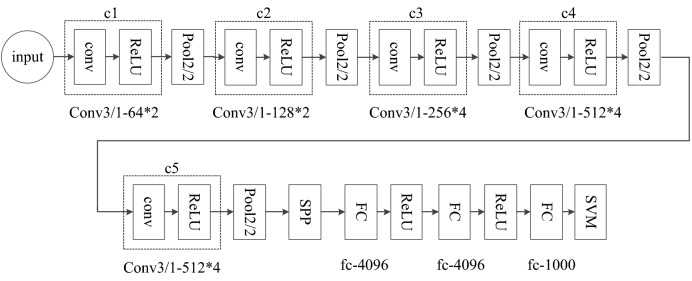
The proposed model

Spatial Pyramid Pooling (SPP) is the process of subpartioning and pooling the extracted image features in a particular pattern thus creating a representation that also preserves spatial information [40]. It can generate feature vectors of fixed size and make the convolution neural network structure adapt to different scale and multi-size image input. Finally the network can effectively extract the multi-scale feature information of samples. In this study, by adding SPP to VGG (Fig. 9), the proposed model can handle any input size, which improves the scale invariance of the image and reduces the over-fitting.

### SVM

In machine learning, SVM is supervised learning model with associated learning algorithms that analyze data used for classification [41, 42]. SVM classifier is a traditional machine learning classification model, which has strong generalization ability. SVM is supported by statistical method theory. It has been widely respected by machine learning scholars. SVM has been widely used in various classification problems and provides top-performing recognition results [10, 21, 22]. In addition, the kernel function method can effectively map the low-dimensional data to the high-dimensional data without increasing the computational complexity. Therefore, SVM can deal with nonlinear problems, thus ensuring that the trained model can provide high accuracy. The advantage of SVM classifier is that it can still achieve better classification results even when there are few samples and high data dimensions.

### Implementation of the VGG-SVM model

**Fig. 10:**
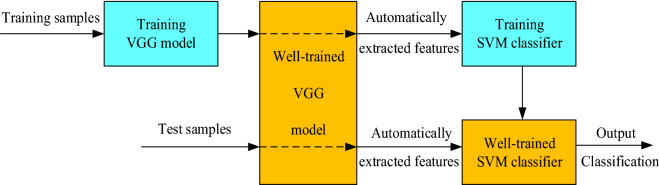
Implementation process of the proposed model

Convolutional neural network has strong adaptability and is skilled at mining local features of data and extracting global training features, but the final classification layer may produce sub-optimal classification plane, which is not the optimal result. For classification problems, most deep learning models employ the softmax function for prediction. In this paper, based on the advantages of the VGG network and SVM classifier discussed in “VGG net” Section and “Spatial pyramid pooling” Section, the architecture of our hybrid VGG–SVM model was designed by replacing the softmax layer of the VGG model with a liner SVM classifier. The VGG network is used to calculate each sample image representation. The SVM classifier can obtain the new high-dimensional feature vectors from the penultimate FC layer of well-trained VGG network for training. Once the SVM classifier has been well trained, we can use the SVM classifier to obtain the results of rape/weeds classification on the testing images, which has the features extracted by well-trained VGG network. The execution process of hybrid VGG-SVM model is shown in Fig. 10, which can be summarized as follows:

1. The images samples are randomly divided into training samples, verification samples and test samples according to a certain proportion.
2. For the training process, the training samples are fed to the input layer of VGG. The training parameters are optimized.
3. After the training of VGG was well done with a number of training samples, the relevant feature vector of each training sample has been automatically extracted by well–trained VGG.
4. Training the SVM classifier with the feature vector of the training samples automatically extracted in the previous step.
5. For the test of SVM classifier, test samples are fed to the well-trained VGG classifier and a test feature vector is obtained.
6. The feature vectors of test samples are classified by well-trained SVM classifier.

## Data Availability

Data and materials are available.
